# Antimicrobial Activity of Bee Venom and Melittin against *Borrelia burgdorferi*

**DOI:** 10.3390/antibiotics6040031

**Published:** 2017-11-29

**Authors:** Kayla M. Socarras, Priyanka A. S. Theophilus, Jason P. Torres, Khusali Gupta, Eva Sapi

**Affiliations:** Lyme Disease Research Group, Department of Biology and Environmental Science, University of New Haven, West Haven, CT 06519, USA; kmsocarras@gmail.com (K.M.S.); priyankaannabel@gmail.com (P.A.S.T.); jtorr3@unh.newhaven.edu (J.P.T.); kgupt2@unh.newhaven.edu (K.G.)

**Keywords:** Lyme disease, bee venom, melittin, biofilms, persisters, antibiotic resistance

## Abstract

Lyme disease is a tick-borne, multi-systemic disease, caused by the bacterium *Borrelia burgdorferi.* Though antibiotics are used as a primary treatment, relapse often occurs after the discontinuation of antimicrobial agents. The reason for relapse remains unknown, however previous studies suggest the possible presence of antibiotic resistant Borrelia round bodies, persisters and attached biofilm forms. Thus, there is an urgent need to find antimicrobial agents suitable to eliminate all known forms of *B. burgdorferi*. In this study, natural antimicrobial agents such as *Apis mellifera* venom and a known component, melittin, were tested using SYBR Green I/PI, direct cell counting, biofilm assays combined with LIVE/DEAD and atomic force microscopy methods. The obtained results were compared to standalone and combinations of antibiotics such as Doxycycline, Cefoperazone, Daptomycin, which were recently found to be effective against Borrelia persisters. Our findings showed that both bee venom and melittin had significant effects on all the tested forms of *B. burgdorferi.* In contrast, the control antibiotics when used individually or even in combinations had limited effects on the attached biofilm form. These findings strongly suggest that whole bee venom or melittin could be effective antimicrobial agents for *B. burgdorferi;* however, further research is necessary to evaluate their effectiveness in vivo, as well as their safe and effective delivery method for their therapeutic use.

## 1. Introduction

Through the years, the severity of infectious diseases and the inability to effectively treat them with antibiotics have become a rapidly growing epidemic. One such disease that has spread across the United States, Europe, Asia, Australia and in some parts of Africa is Lyme borreliosis, alternatively known as Lyme disease [1,2]. The known causative agent of Lyme disease is *Borrelia burgdorferi*, which is transmitted primarily through Ixodid ticks [1,3]. According to the Center of Disease Control, the United States has approximately 300,000 newly reported Lyme disease cases every year [4]. Successfully diagnosed individuals are often prescribed antibiotics such as Doxycycline, Amoxicillin and Ceftriaxone; however, recent studies demonstrated that these antibiotics are insufficient in eliminating certain forms of *Borrelia* spp. in vitro and in vivo [5,6,7,8,9,10,11,12,13].

*Borrelia* spp., by its traditional definition, is a spirochetal bacterium with internalized flagella [14,15], however, other morphological forms were also identified such as round bodies, stationary phase persisters and biofilm forms [16,17,18,19,20,21,22]. *B. burgdorferi* can transform between these morphologies depending on its environment [23]. Some factors that cause these different forms are certain unfavorable conditions such as changes in pH, nutrient starvation, host immune system attacks, or even antibiotics could promote these morphological changes [16,17,20,22,24]. These defensive forms were reported to have high resistance to the antimicrobials agents that are currently used to treat Lyme disease (7, 21, 22). For example, while Doxycycline is very effective eliminating spirochetes in vitro, it did not reduce antibiotic resilient persisters and/or biofilms [6,7,22,25]. Furthermore, it was demonstrated that none of the antibiotics currently used to treat Lyme disease effective against the “persister” and attached biofilm forms of *Borrelia* [7,8,9,10,22,25,26]. It was also reported that several antibiotics (Cefoperazone, Daptomycin) might have potential in effectively eliminating *Borrelia* persisters especially when in combination with Doxycycline [8,10]. Unfortunately, attached *Borrelia* biofilms, which were recently proven to be present in infected human skin tissues, did not respond well to these new antibiotic combinations [26].

Considering the limiting effects that standard antibiotics may have on the Borrelial morphologies, our research group began searching for potential alternative antimicrobials. In a recent study, *Stevia rebaudiana* leaf extract was found to be very effective in eliminating all known Borrelia morphological forms including attached biofilms [26]. Based on these findings, we looked for additional alternative agents that may also have similar effect. One alternative agent is apotoxin—also known as bee venom—derived from the insect *Apis mellifera* better known as the honeybee. The use of this venom has been documented for its medicinal purposes for approximately 6000 years ago and several studies have proven its antimicrobial effects [27,28]. In a previous study, bee venom’s component melittin was shown to have significant effects on *Borrelia* spirochetes at MIC concentrations of 100 μg/mL [29]. Recent data shows similar MIC values for melittin when used to treat several other gram-negative microorganisms such as *Salmonella enterica* and *Yersinia kristensenii* [30]. In this report, we expanded these findings by testing the sensitivity of different forms of *B. burgdorferi* to bee venom and its component melittin in comparison to antibiotics recently found effective against Borrelia persister forms [7,8,9,10]. To assess antimicrobial sensitivity of bee venom and melittin, previously published methods such as SYBR Green I/PI assay combined with total direct live cell counting were used for log phase spirochetes and stationary phase persisters [6,31], while attached biofilms were analyzed by crystal violet and LIVE/DEAD staining techniques [6]. Fluorescent and atomic force microscopy methods were also employed to further visualize the effect of these antimicrobial agents on Borrelia.

## 2. Results

Prior to testing the potential antimicrobial effect of bee venom on *B. burgdorferi* using SYBR Green I/PI assay, bee venom, melittin and all the antibiotics used in this study were analyzed for auto fluorescence due to reported findings of potential auto-fluorescence issues of certain antimicrobials in previous studies [24,29,31]. Values from auto fluorescence detected from any of the antimicrobials were deducted from future experiments. However, due to reports of cellular auto fluorescence from antibiotic treated bacterial cells following intracellular damage [32] all results from the SYBR Green I/PI were confirmed using the direct counting method of live/dead cells using the Live/Dead assay [6,7,8,9,10,26,31].

**Figure 1:**
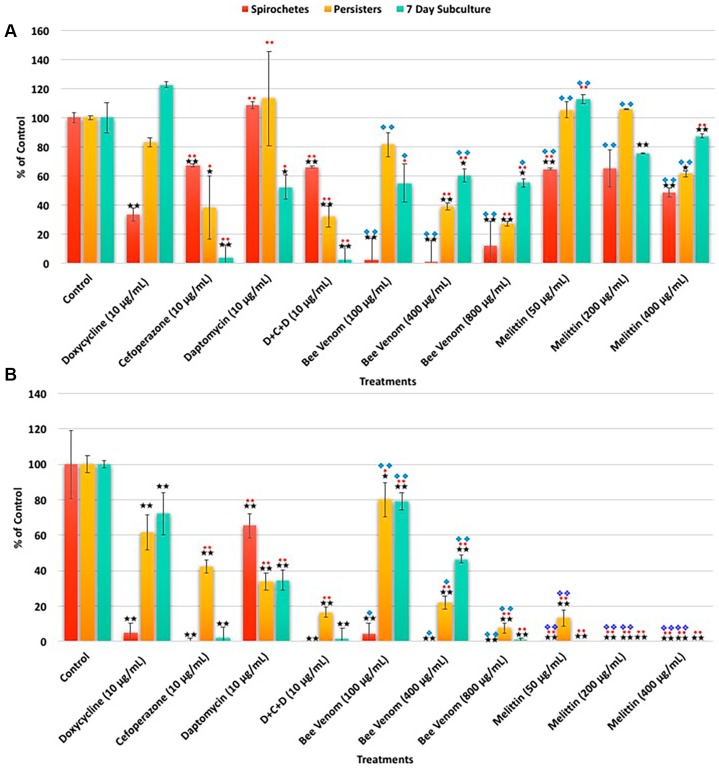
The effects of various antimicrobial agents on *B. burgdorferi* as determined by SYBR Green I/PI assay Panel (**A**) or direct counting assay Panel (**B**). Doxycycline, Cefoperazone, Daptomycin and their combination (D + C + D) as well as different concentrations of bee venom and melittin were tested on *B. burgdorferi* logarithmic phase (spirochetes) culture and stationary phase (persisters) cultures as well as in 7-day recovery subculture as described previously [6,7,8]. Significance against sterile PBS buffer (control vehicle) with the *p* value of <0.05 and <0.01 are indicated in * and ** respectively. Significance against Doxycycline with the *p* value of <0.05 and <0.01 are indicated in ♦ and ♦♦ respectively. Significance against the three-antibiotic combination (D + C + D) with the *p* value of <0.05 and <0.01 are indicated in ❖ and ❖❖ respectively. *n* = 9.

In the first set of experiments, antibiotics recently reported to be effective for several *Borrelia* forms were tested to confirm the previous findings [31]. Doxycycline, Cefoperazone, Daptomycin, as well as the combination of the three-antibiotics (D + C + D), were used in concentrations reported effective on both logarithmic phase (spirochetes) and stationary phase (persisters) cells of *B. burgdorferi.* To determine the long-term effects of all antimicrobials, recovery cultures were used in which treated cells were further cultured in antibiotic free media for 7 days as described previously [7,8,9,10,26,31]. Furthermore, because our previous research shows that the free floating and surface bound aggregate forms could have different antibiotic sensitivity [6,26], we separately studied the effect of bee venom and melittin on the surface attached biofilm form as well as the free-floating aggregates. As a negative control, appropriate amounts of sterile PBS buffer were used in all experiments. To determine the effectiveness of the different antimicrobials, SYBR Green I/PI assay and direct counting methods were used in parallel and results are depicted in Figure 1A,B respectively. Data generated from both methods were in good agreement to previously reported data for all the antibiotics tested [31], i.e., Doxycycline significantly reduced the number of spirochetes but not the persisters (Figure 1A). In contrast, Cefoperazone, Daptomycin and the three-antibiotic combination (D + C + D) significantly reduced viable spirochetes and persisters (Figure 1A). However, results from SYBR Green I/PI assay and direct counting were significantly different. The direct counting method indicated a higher cell death rate for Daptomycin and the three-antibiotic combination (D + C + D) treatments (Figure 1A,B respectively).

Furthermore, due to the unknown half-life of the potentially active components in bee venom on Borrelia, we compared the effect of bee venom administered in both a single treatment and daily regimens for 3 days (Figure 1 and Figures S1–S4, Table S1 in Supplementary Materials). The daily treatment protocol was found to be significantly more effective and is shown in Figure 1 and used in future experiments.

When SYBR Green I/PI assay was used, bee venom data showed that the number of viable logarithmic phase spirochetes were significantly lower at all concentrations (*p* value ≤ 0.01) than the PBS treated negative control or any of the antibiotic treated cultures except Doxycycline (*p* value ≤ 0.01) (Figure 1A). When bee venom was used at a concentration above 100 μg/mL, bee venom, the results demonstrated a greater reduction in spirochetes than the Doxycycline treated cultures. Stationary phase persisters treated with bee venom concentrations above 100 μg/mL were significantly reduced compared to the negative control and Doxycycline (*p* value ≤ 0.01) (Figure 1A). In addition, the effect of bee venom treatment was comparable to Cefoperazone and the three antibiotics combination (D + C + D) at concentrations at greater than or equal to 400 μg/mL (Figure 1A). In the 7-day subculture experiments testing for cells, which were able to recover after antimicrobial treatments, there was a significant decrease (*p* value ≤ 0.01) in the number of viable cells compared to the negative control and Doxycycline (*p* value ≤ 0.01).

In parallel experiments, one of the major antimicrobial components of bee venom, melittin, was tested first using SYBR Green I/PI assay (Figure 1A). Bee venom is comprised of 50% melittin, therefore the concentrations used for testing melittin were 50% less than when whole bee venom was used. Melittin was administered daily at concentrations shown previously to have significant effect on Borrelia spirochetes [29]. Results from this study showed that melittin could significantly decrease the numbers of persisters (*p* value ≤ 0.05) compared to the negative control (Figure 1A). Melittin, at concentrations below 400 μg/mL however, showed significantly higher viable spirochete numbers (*p* value ≤ 0.05) than Doxycycline (*p* value ≤ 0.01). In the 7-day recovery subculture there were significantly fewer cells (*p* value ≤ 0.01) when concentrations above 200 μg/mL of melittin were used compared to the negative control and Doxycycline (Figure 1A). Melittin was significantly less effective on recovered subculture cells (*p* value ≤ 0.01) in comparison to the three-antibiotic combination treatment (D + C + D).

To confirm *B. burgdorferi* viability after bee venom and melittin treatment, all SYBR Green I/PI assay results were confirmed via a total viability direct counting method as described previously [6,7,8,26]. In these experiments, a significant reduction of viable cells was found when exposed to whole bee venom at all concentrations compared to the negative control and Daptomycin (*p* value ≤ 0.01) (Figure 1B). Similarly, exposure with concentrations >100 μg/mL of whole bee venom resulted in significantly fewer persisters in comparison to the negative control, Doxycycline and Cefoperazone (*p* value ≤ 0.01) in a dose-dependent manner (Figure 1B). The 7-day subculture experiments showed that doses above 400 μg/mL of bee venom effectively eliminated live cells similarly to Cefoperazone and to the three-antibiotic combinations (D + C + D) (Figure 1B).

When the effectiveness of melittin on *B. burgdorferi* was tested using a direct counting approach, there was again a significant difference between the results from the SYBR Green I/PI assay and total direct counting method (Figure 1B). Results showed that melittin significantly reduced the numbers spirochetes (*p* value ≤ 0.01) at all concentrations compared to the negative control and Doxycycline (*p* value ≤ 0.01) (Figure 1B). Persisters treated with melittin showed significant reduction at all concentrations (*p* value ≤ 0.01) compared to the negative control, Doxycycline and the three-antibiotic combination (D + C + D) (Figure 1B). The 7-day recovery subcultures exposed to all concentrations of melittin were also significantly reduced (*p* value ≤ 0.01) compared to the negative control, bee venom and all used antibiotics (Figure 1B). In summary, we concluded that the MIC concentration of bee venom on Borrelial spirochetes is 200 μg/mL and the MIC for melittin is 100 ug/mL.

**Figure 2:**
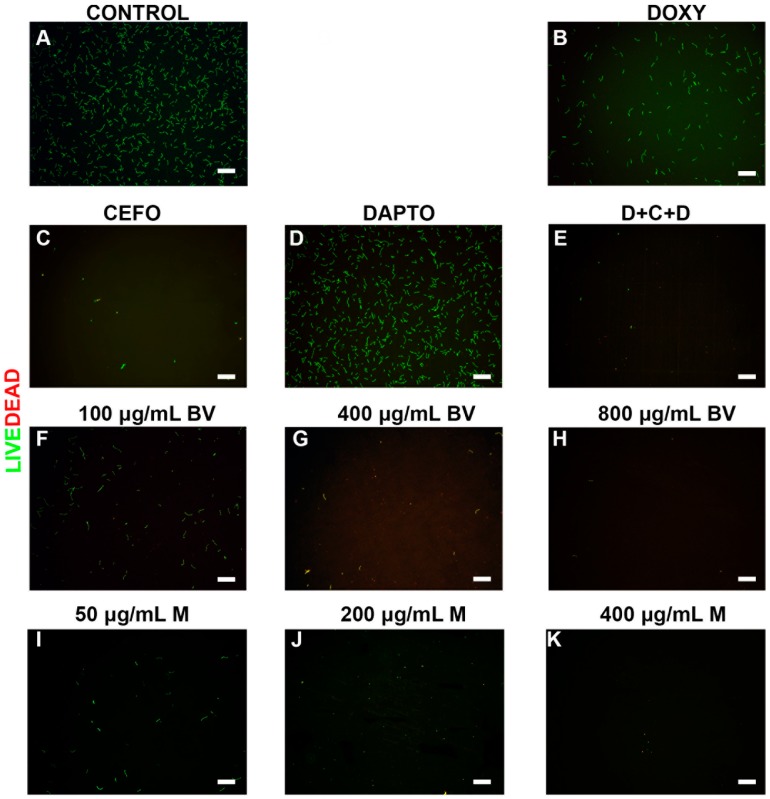
Representative Live/Dead staining images of *B. burgdorferi* log phase spirochetal cultures treated with different antimicrobial agents. Cells were stained with SYBR Green I/PI as outlined in the Methods and representative images were taken at 100× magnification. Panel (**A**) Borrelia culture treated only with PBS was used as a negative control. Panel (**B**) Doxycycline (DOXY) treated; Panel (**C**) Cefoperazone (CEFO) treated; Panel (**D**) Daptomycin (DAPTO) treated and Panel (**E**) Three-antibiotic combination (D + C + D). Panels (**F**–**H**) Bee venom (BV) was used in increasing concentrations while Panels (**I**–**K**) depicts melittin (M) treated cells. Live cells are stained with green color while dead cells are stained red. Scale bar: 100 μm.

**Figure 3:**
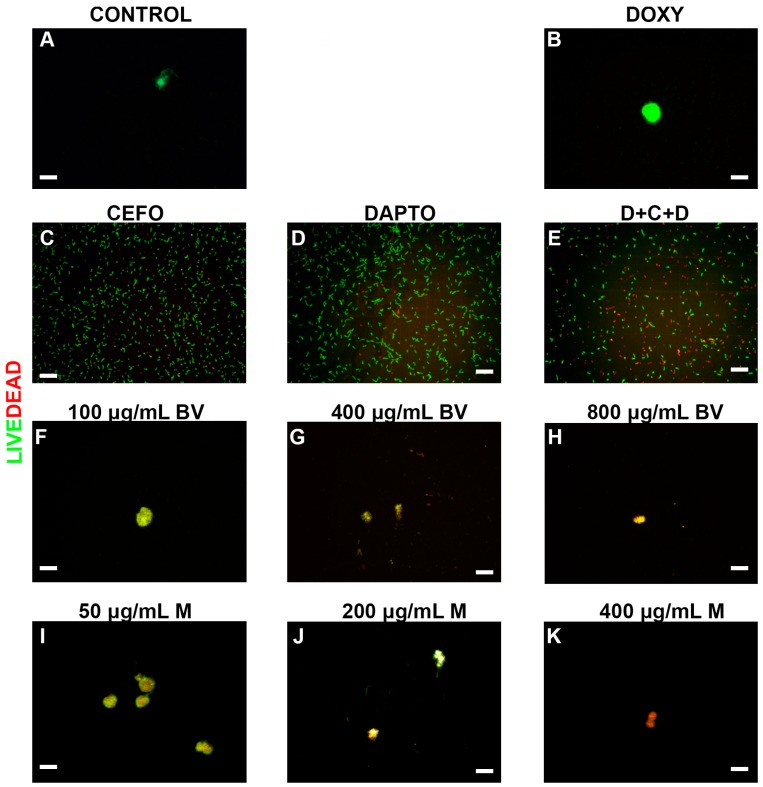
Representative Live/Dead staining images of *B. burgdorferi* stationary phase persister cultures following treatment with different antimicrobial agents. Cells were stained with SYBR Green I/PI as outlined in the Methods and representative images were taken at 100× magnification. Panel (**A**) Borrelia culture treated only with PBS was used as a negative control. Panel (**B**) Doxycycline (DOXY) treated, Panel (**C**) Cefoperazone (CEFO) treated, Panel (**D**) Daptomycin (DAPTO) treated and Panel (**E**) Three-antibiotic combination (D + C + D). Panels (**F**–**H**) Bee venom (BV) was used in increasing concentrations while Panels (**I**–**K**) depicts melittin (M) treated cells. Live cells are stained with green color while dead cells are stained red. Scale bar: 100 μm.

**Figure 4:**
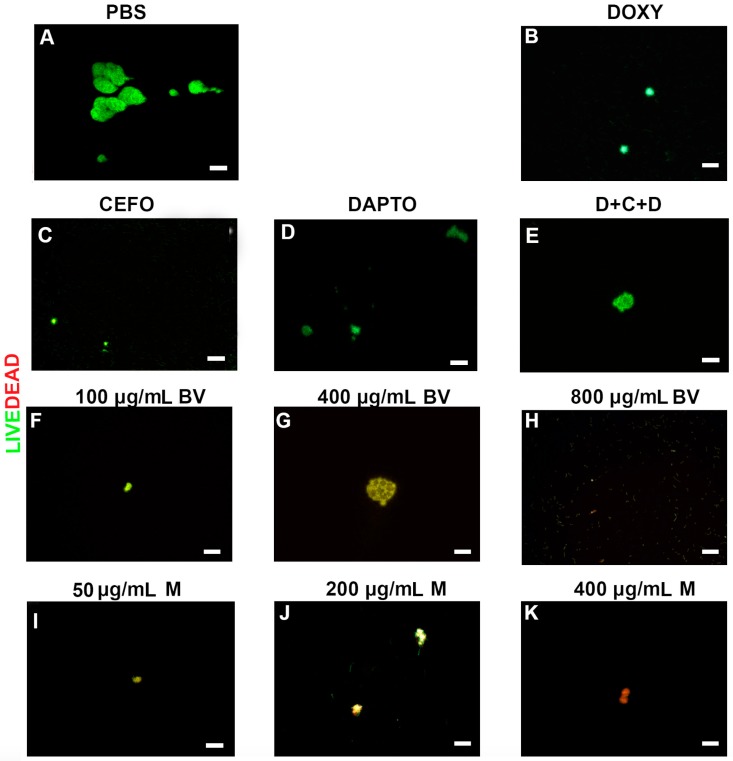
Representative Live/Dead staining images of *B. burgdorferi* 7-day recovery cultures following treatment with different antimicrobial agents. Cells were stained with SYBR Green I/PI as outlined in the Material and Methods and representative images were taken at 100× magnification. Panel (**A**) Borrelia culture treated only with PBS was used as a negative control. Panel (**B**) Doxycycline (DOXY) treated, Panel (**C**) Cefoperazone (CEFO) treated, Panel (**D**) Daptomycin (DAPTO) treated and Panel (**E**) Three-antibiotic combination (D + C + D). Panels (**F**–**H**) Bee venom (BV) was used in increasing concentrations while Panels (**I**–**K**) depict melittin (M) treated cells at different concentrations. Live cells are stained with green color while dead cells are stained red. Scale bar: 100 μm.

To further verify the effectiveness of all antimicrobial agents, the viability of the different morphological forms and cultures of *B. burgdorferi* were evaluated by a LIVE/DEAD staining method combined with fluorescent microscopy imaging. Representative images visualize the effects of these treatments on spirochetes (Figure 2), persister cells (Figure 3) and 7-day recovery subcultures (Figure 4). Panel As in Figure 2, Figure 3 and Figure 4 depict the negative control (PBS control), while positive controls (antibiotics and antibiotic combinations) were shown in panels (B–F) in Figure 2, Figure 3 and Figure 4. For the individual antibiotics and the antibiotic combination, the obtained images were in agreement with the direct counting data. For example, Cefoperazone and three-antibiotics combination (D + C + D) were very effective in eliminating spirochetes, a data which was found by the direct counting data but not with SYBR Green I/PI method (Figure 1A,B).

*B. burgdorferi* cultures treated with various concentrations of bee venom were shown in the subsequent panels (G–I) (Figure 2, Figure 3 and Figure 4), while cultures treated with melittin were shown in panels (J–L) (Figure 2, Figure 3 and Figure 4). As previously mentioned, results from these images supported the total direct count numerical data but not the SYBR Green I/PI assay. For example, bee venom exposure showed a significant decrease in the number of spirochetes, persisters, as well as 7-day recovery subculture in comparison to the negative and positive controls (Panels (G–I) in Figure 2, Figure 3 and Figure 4). Similarly, melittin treatment demonstrated a dramatically significant decrease in live cell numbers for both log phase, stationary phase and recovery cultures, which agreed with the direct counting data (Panels (J–L), Figure 2, Figure 3 and Figure 4). Table 1 summarizes the effects of various antimicrobial agents on *B. burgdorferi* as determined by SYBR Green I/PI (Panel (A)) or direct counting assays (Panel (B)).

**Figure 5:**
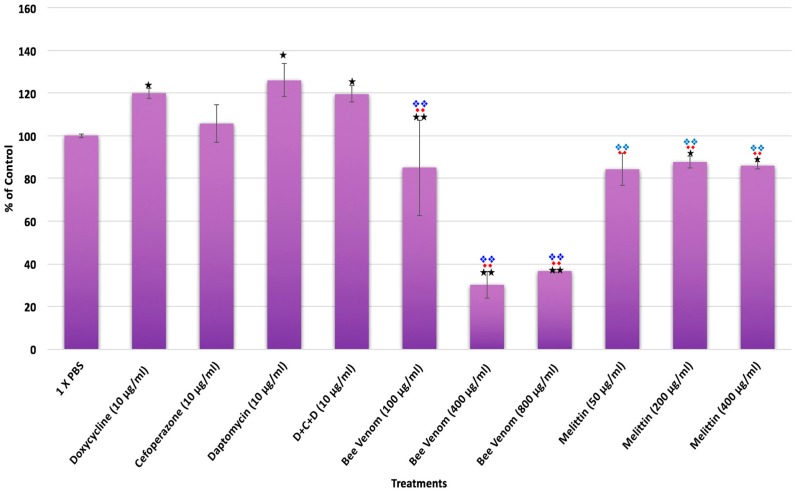
Effect of different antimicrobial agents on attached *B. burgdorferi* biofilms. Susceptibility of attached *B. burgdorferi* biofilms to antimicrobial agents after a three-day treatment was analyzed by crystal violet method as described in Material and Methods. Doxycycline, Cefoperazone, Daptomycin and their combination (D + C + D) as well as different concentration of bee venom and melittin were tested on attached Borrelia biofilms. Significance against PBS buffer (negative control vehicle) with the *p* value of <0.05 and <0.01 are indicated in * and ** respectively. Significance against Doxycycline with the *p* value of <0.05 and <0.01 are respectively indicated in ♦ and ♦♦ Significance against the three-antibiotic combination (D + C + D) with the *p*-value of <0.05 and <0.01 are indicated in ❖ and ❖❖ respectively. *n* = 9.

**Figure 6:**
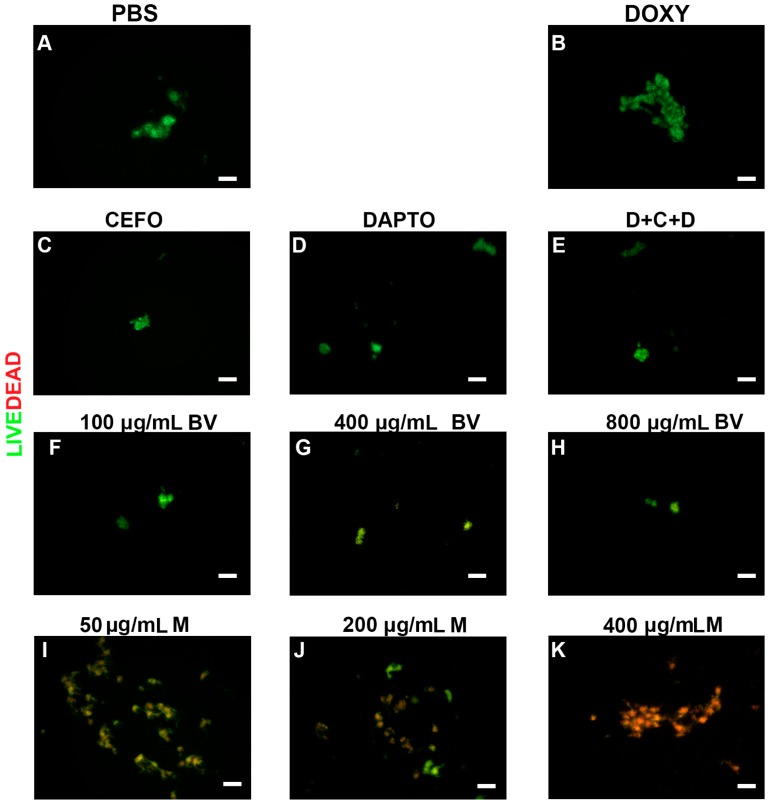
Representative Live/Dead images of the viability of attached Borrelia biofilms following treatment with different antimicrobial agents. Biofilms were stained with SYBR Green I and PI as outlined in the Material and Methods and representative images were taken at 100× magnification. Panel (**A**) Borrelia culture treated only with PBS was used as a negative control. Panel (**B**) Doxycycline (DOXY) treated, Panel (**C**) Cefoperazone (CEFO) treated, Panel (**D**) Daptomycin (DAPTO) treated and Panel (**E**) Three-antibiotic combination (D + C + D). Panels (**F**–**H**) Bee venom (BV) was used in increasing concentrations while Panels (**I**–**K**) depict melittin treated cells at different concentration. Live cells are stained with green color while dead cells are stained red. Scale bar: 100 μm.

In the past several years, a novel aggregate form, called biofilm, was found for Borrelia and was shown to be very antimicrobial resistant vitro and in vivo especially in attached forms [6,22,25,26,33]. Therefore, in subsequent experiments we tested all antimicrobials for effectiveness in eliminating the attached biofilm form. To evaluate the effect of antimicrobial agents on the attached Borrelia biofilms, first we used crystal violet quantitative biofilm assay as described previously [6,26]. As negative control, the appropriate amounts of PBS were used (control vehicle) and all presented data were normalized to the negative control. Bee venom exposure at >100 μg/mL but none of the antibiotics or their combination (D + C + D), significantly reduced Borrelia biofilm mass (*p* value ≤ 0.01) in comparison to the negative control (Figure 5). Melittin also reduced Borrelia biofilm mass at different concentrations compared to the negative control (*p* value ≤ 0.05), or Doxycycline (*p* value ≤ 0.01), or the three-antibiotic combination (D + C + D; *p* value ≤ 0.01) but were found less effective than whole bee venom at >100 μg/mL (Figure 5). To verify these findings, LIVE/DEAD staining method combined with fluorescent microscopy imaging was used. Representative microscopy images confirmed the decreased size of Borrelia biofilm with the bee venom (Figure 6, panels (F–H)) and melittin treatment (Figure 6, panels (J–L)). Interestingly, melittin also significantly reduced biofilm viability (red stain Figure 6, panels (I–K)). Some of the antibiotics (Cefoperazone and Daptomycin and the three antibiotic combination (D + C + D); Figure 6, panels (C–E) respectively) also showed some reduction in biofilm sizes, which were not detected with the crystal violet assay; however, those remaining biofilms were stained green suggesting that they are viable (Figure 6, panels (A–J)).

**Figure 7:**
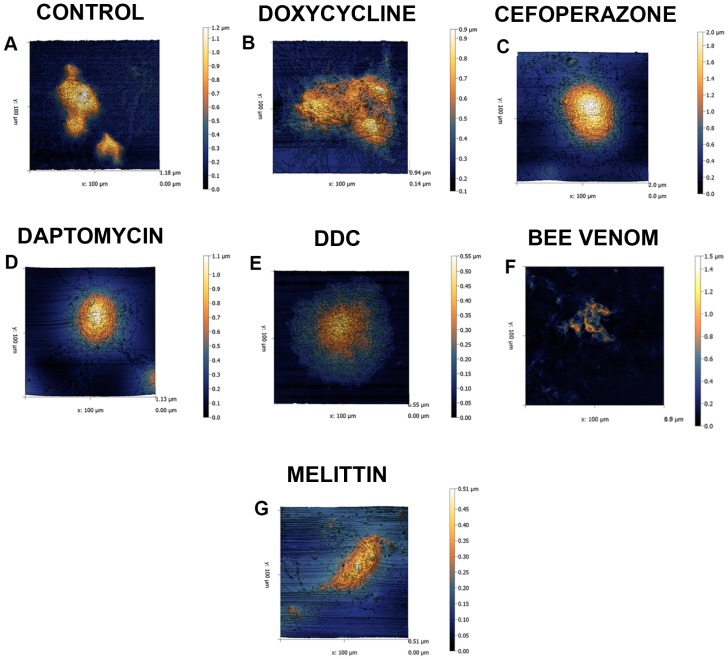
Representative atomic force microscopy images showing the ultrastructural details of Borrelia biofilm before and after treatment with antimicrobial agents. The preparations of *B. burgdorferi* strain B31 biofilms on chamber slides are described in Methods section. All biofilms were scanned at 0.4 Hz using contact mode and the individual Z ranges (height) are indicated next to each panel by means of a scale. The images were scanned using the Nanosurf Easyscan 2 software and the images were processed using Gwyddion software. Scale bar located on the side of corresponding AFM scan indicate the height changes of the topography of the biofilm. Darker colors (black and blue) indicate the surface of the slide while lighter colors (yellow to white) indicate high points of attached biofilms. Attached Borrelia biofilms treated with Panel (**A**) PBS (control), Panel (**B**) Doxycycline (10 μg/mL), Panel (**C**) Cefoperazone (10 μg/mL), Panel (**D**) Daptomycin (10 μg/mL), Panel (**E**) Three antibiotic combination: Doxycycline + Daptomycin + Cefoperazone (DCC, 10 μg/mL/each), Panel (**F**) Bee venom (400 μg/mL) and Panel (**G**) Melittin (200 μg/mL).

Finally, the ultrastructure of attached *B. burgdorferi* biofilms treated with different antimicrobials were studied using atomic force microscopy. In these experiments, attached Borrelia biofilms were exposed to the different antimicrobials as described above then analyzed for changes in topography and size (Figure 7). The atomic force microscopic images are 3D rendered and digitally colored for improved visualization (Figure 7). The negative control was shown in Figure 7 Panel (A) followed by the positive controls in Panels (B–E). Subsequently, the effects of bee venom and melittin were shown in Panels (F) and (G) respectively. The drug-free control had a very compact and rigid structure (Figure 7, Panel (A)) similarly to the biofilms treated with Doxycycline, Cefoperazone, Daptomycin and three-antibiotic combination (D + C + C) respectively (Figure 7, Panels (B–E)). Bee venom and melittin treated biofilms however, revealed a very loose structure suggesting the effectiveness of those agents against biofilm structure (Figure 7, Panel (F,G)).

## 3. Discussion

The spirochetal bacterium *B. burgdorferi* sensu lato is the main pathological agent of Lyme disease in North America and Europe [1,2]. While this infectious disease may be treated with antibiotics, there has been a rise in antibiotic resistance in recent years [34]. Therefore, extensive effort has been made in finding novel antimicrobial compounds that can assist in the treatment of Lyme disease. In this study, whole bee venom, as well as its component, melittin, were tested on different forms of Borrelia. This idea was based on a promising earlier study, which showed that melittin significantly affected *B. burgdorferi* sensu stricto spirochetes by decreasing the bacterium’s motility as well as its growth [29]. The effects of bee venom and melittin were compared against antibiotics from a study that used the FDA drug library to find highly effective agents for *B. burgdorferi* [7]. The study also confirmed several previous findings that not all antibiotics being used for Lyme disease treatment are effective on all morphological forms of *B. burgdorferi* [6,7,8,26]. A later study showed that newly discovered antibiotic combinations that were effective for Borrelia persisters [7,8,9,10] had limited effect on attached Borrelia biofilms [26]. Therefore, our study aimed to evaluate whether bee venom and melittin could be effective for all morphological forms of Borrelia.

We utilized several different methods to evaluate the effect of all antibiotics and antimicrobials and found that certain techniques such as SYBR Green I/PI needed to be confirmed by additional assays. A potential explanation for the findings is that the SYBR Green I/PI assay could be affected by auto fluorescent components of the dying cells as previously reported for *Escherichia coli* treated with antibiotics [32]. It was suggested that cell death could trigger changes in intrinsic cellular constituents and produce fluorescent chemical compounds [35].

Based on findings from different techniques, we concluded that both whole bee venom and melittin could have significant effects on all Borrelia morphological forms including inhibiting the recovery of spirochetal cells and persisters as evidenced by recovery cultures in antimicrobial free media. Whole bee venom and melittin also significantly reduced the number and/or viability of attached biofilms, which based on previous research, is the most antibiotic resistant form of *B. burgdorferi* [6,25,26]. The MIC concentration values for melittin, for example, were in good agreement with previous studies that evaluated melittin on Borrelia spirochetes [29] and on several other gram-negative microorganisms such as *S. enterica* and *Y. kristensenii* [30].

Comparison of the observed effects of whole bee venom and melittin on Borrelia showed some differences, however. For example, ultrastructure analyses using atomic force microscopy revealed that whole bee venom treatment had more of an effect on the morphology and size of the biofilms than its viability, suggesting the complexity of biofilm responsiveness to antimicrobial agents, which requires further investigation. Differences in bee venom and melittin effectiveness on *B. burgdorferi* suggest that there may be other components within the whole bee venom, that also have an effect on Borrelia biofilms. Similar findings were reported by a recent study testing different whole leaf Stevia extracts on *B. burgdorferi* [26], which found that while whole Stevia leaf extracts were effective, its known component stevioside was not. The results suggested that other components within the whole Stevia leaf extract might affect Borrelia either individually or in a potentially synergistic capacity with stevioside. In addition, the standard antibiotics used in this study had little or no effect on attached biofilm forms of *B. burgdorferi*. Similar findings were observed in previous studies on *Pseudomonas aeruginosa* and *Staphylococcus aureus*, which found that antibiotics could not eliminate the biofilm form and in some cases, could even increase its size [36,37,38,39]. In our study, a similar result was found for Doxycycline; it actually increased the attached Borrelia biofilm mass, an observation that agreed to previously published findings [6,25,26].

Bee venom has been shown in past studies to have a wide range of applications in reducing or even eliminating ailments [28,40,41,42,43,44] which can be explained by the multitude of components of bee venom that give it its properties. One type of component, called antimicrobial peptides, could not just eliminate pathogens but could also affect inflammation, enhanced would healing and even had anti-biofilm behavior on different microorganisms [30,42,45,46,47,48,49]. Furthermore, these specific peptides could also affect the bacteria’s ability to create fully functional biofilms [50]. Thus, it is vital to understand the components used within the study to comprehend its significance as an antimicrobial treatment.

Melittin is a small, amphipathic α–helical antimicrobial peptide of 26 amino acids and comprises approximately 50% of the whole bee venom used within our study [40,41,43,44,51]. The antimicrobial peptides in bee venom, including melittin and phospholipase A_2_, have been a topic of interest within the scientific community, due to the versatility in its function in innate immunity, as well as minimizing chances of adverse immunological reactions when used in combination with other compounds. In this study, Phospholipase A_2_ was also tested at different concentrations but did not show any significant effect on any of the morphological forms of *Borrelia* (data not shown).

In recent years, there has been a focus on melittin and its mechanism of action for targeting different microbes [27,42]. This antimicrobial peptide, similar to most of its kind, is amphipathic. This allows for melittin integration into target phospholipid bilayers in low concentrations, while in high concentrations it homodimerizes to form pores, releasing Ca^2+^ ions or disrupting phospholipid head groups [27,38,52,53,54]. In *B. burgdorferi,* the Ca^2+^ ions are used for the development of a protective outer shell for mature biofilms, for the evasion of potential host resistance [22,25]. The specific mechanism of action of melittin, much like other antimicrobial peptides, are dependent on the target bacteria’s phospholipid bilayer composition, as well as evasion of common antimicrobial treatments which could affect the binding locations of peptides to the cell membrane [45,54,55].

Antimicrobial peptides are well known to have very high activity towards microbial membranes with low antimicrobial resistance development [48,56,57,58]. One of the important reasons that biofilm structure could provide high resistance to antibiotics is mainly due to the presence of dormant microbial populations (sleepers) inside the biofilms. These biofilms are very difficult to kill with standard antibiotics, which often relies on actively growing cells [59]. The use of certain antimicrobial peptides such as mellittin could eliminate this problem by permeabilizing microbial membranes, which results in membrane disruption and cell death even for those dormant cells in the center of the biofilm [48,49]. Interestingly, however, recent findings indicate that antimicrobial peptides can also have intracellular targeting that affects nucleic acid and/or protein synthesis even protein foldings [60]. However future studies are necessary to evaluate whether this is true for melittin.

Another focus in recent publications on bee venom or melittin is the clinical effectiveness of these natural antimicrobials on different diseases. Melittin, for example, was shown to have a very strong immunoregulatory activity, anticancer effect and even shows promise as chemotherapy of human immunodeficiency virus (HIV) infection [41,43,61,62]. Unfortunately, several issues were raised as to the safe administration of melittin in the clinical setting, due its cytotoxicity to human cells; for example, it has the ability to lyse human erythrocytes, exhibits necrotic activity against gastrointestinal and vaginal epithelial cells and can trigger severe allergic reactions [43,63,64,65]. To reduce it cytotoxic affects, recent studies showed that melittin could be paired with various pharmaceutical agents to specifically eliminate cancer cells, which led to further promising clinical trials [51,66]. In another effort of reducing melittin cytotoxicity, melittin was bound to a nanoparticle, which protected normal human cells while it efficiently attacks HIV infected cells (Hood et al., 2013). The findings from these studies could help promote the design of a novel approach for the successful application of bee venom or melittin in the treatment against *B. burgdorferi,* as well as other pathogenic microbes.

## 4. Materials and Methods

### 4.1. Bacterial Culture

*Borrelia burgdorferi* strain B31 was obtained via American Type Culture Collection (ATCC, #35210). Bacteria were maintained at low passage isolates (≤4) in Barbour-Stoner-Kelly H (BSK-H) media (Sigma, St. Louis, MO, USA) supplemented with 6% rabbit serum (Pel-Freez Biologicals, Rogers, AR, USA) free from antibiotics in sterile glass 15 mL tubes and incubated at 33 °C with 5% CO_2_.

### 4.2. Antimicrobial Agent Preparation

*Apis mellifera* venom for in vitro testing and prepared using sterile 1× phosphate buffer saline pH 7.4 (PBS) (Fisher, Waltham, MA, USA). Natural melittin extracted from bee venom was purchased (Sigma, St. Louis, MO, USA) and prepared for testing as directed by the manufacturer. The antibiotics (Doxycycline, Cefoperazone, Daptomycin) were purchased from Sigma and prepared at 10 mg/mL stock per manufacturer’s instructions. In addition, Doxycycline, Cefoperazone and Daptomycin were also combined (D + C + D) to test on *B. burgdorferi* for the treatment of persister cells. All antimicrobial agents were sterilized using a 0.1 μm filter unit (Millipore, Billercia, MA, USA), aliquoted and stored at −20 °C before further use.

### 4.3. Antimicrobial Testing

#### 4.3.1. Bacterial Preparation

Antimicrobial treatment effectiveness was tested on logarithmic phase and stationary phase of *B. burgdorferi* spirochetes using SYBR Green I/PI assay and direct counting method [6,26,31]. Spirochetes in logarithmic phase were seeded at 1 × 10^5^ cells/mL on 96-well sterile tissue culture plates (BD Falcon, Frankline Lakes, NJ, USA) then incubated for 48 h prior to antimicrobial treatment. Stationary phase cultures were seeded at 5 × 10^6^ cells/mL in a 96-well sterile tissue culture plate for 5 days prior to treatment. Spirochetes for surface attached biofilms were seeded at 5 × 10^6^ cells/mL in 4-well Permanox chamber slides (Thermo Scientific, Waltham, MA, USA) for 5 days to establish biofilm form. Floating spirochete cells and aggregates from the supernatant were removed to ensure only surface attached biofilms will be analyzed.

#### 4.3.2. Subculture Experiments

Experiments were prepared with a 1:75 dilution of antimicrobial treated stationary culture placed into antimicrobial agent free media and incubated for 7 days using standard culture conditions. Following incubation, the viability was assessed using the SYBR Green I/PI assay and direct counting method as described below.

#### 4.3.3. SYBR Green I/Propidium Iodide Assay

To analyze antimicrobial agent effectiveness, a standard SYBR Green I/Propidium Iodide assay (SYBR Green I/PI) was performed as previously described [26,31]. Staining mixture was prepared using sterile nuclease free water (Fisher, Waltham, MA, USA), SYBR Green I (10,000× stock, Invitrogen, Grand Island, NY, USA) and propidium iodide (20 mM, Thermo Scientific) before being used on *B. burgdorferi* samples. Stained culture was incubated in the dark for 15 min on a rocking platform before being measured on a fluorescent reader (BioTek FL×800) at 485 nm (setting excitation), the absorbance wavelength at 535 nm (green emission) and 635 nm (red emission). Standard curves were generated for spirochetes, persisters and 7-day subculture cells by preparing live:dead samples. Dead cells were prepared by adding 70% isopropyl alcohol for 15 min (Fisher Scientific, Waltham, MA, USA) while live cells were left untreated. To generate a standard curve, different ratios of live and dead cell suspensions (live:dead ratios = 0:10, 2:8, 5:5, 8:2, 10:0) were added to the wells of the 96-well plate and stained as aforementioned. Using least square fitting analysis, the regression equation was calculated between the percentage of live bacteria and green/red fluorescence ratios. The regression equation was used to calculate the percentage of live cells in each sample of the screening plate. Also, images of the treated sample were taken using fluorescent microscopy (Leica DM2500, Leica Microsystems, Inc. Buffalo Grove, IL, USA) at 100× magnification.

#### 4.3.4. Direct Viable Cell Counts of *B. burgdorferi*

As a confirmation test, the SYBR Green/PI stained cultures were assessed for cell growth by directly counting live and dead bacteria using a bacterial counting chamber (Hausser Scientific, Horsham, PA, USA) using fluorescent microscopy (Leica DM2500). As above, using least square fitting analysis the regression equation was calculated and used to calculate the percentage of live cells in each sample.

#### 4.3.5. Autofluorescence of Antimicrobials

All antimicrobial agents were tested for auto fluorescence due to previously reported issues in SYBR Green I/PI assay for detection potential auto fluorescence of the agents [7,26]. In a 96-well plate, antimicrobials were tested in 100 μL of BSK-H media using the SYBR Green I/PI assay and the obtained auto fluorescence values were subtracted from the obtained experimental values.

#### 4.3.6. Quantitative Assay for Attached Biofilms.

The efficacy of antimicrobial agents on attached biofilms were quantified by measuring the total biomass using crystal violet staining before and after antimicrobial treatments. All centrifugation steps were performed at 12,000× *g* at room temperature for 5 min. At the end of the treatment regimen, culture media was discarded and attached biofilms were collected by adding 500 μL of 1× PBS before being pelleted. Supernatant was discarded and 50 μL of (0.01% *w*/*v*) crystal violet (Sigma, St. Louis, MO, USA) was added to biofilms prior to a 10-min incubation at room temperature. Unbound stain was removed by centrifugation before the biofilm pellet was washed with non-sterile 1× PBS and re-centrifuged. The resulting supernatant was discarded and 200 μL of 10% acetic acid (Sigma, St. Louis, MO, USA) was added to the pellet to release and dissolve excess crystal violet stain during a 15-min incubation period at room temperature. Following incubation, the biofilms were centrifuged and the remaining crystal violet staining was extracted, transferred to a 96-well plate and read at 595 nm using a BioTek Spectrophotometer (BioTek, Winooski, VA, USA).

### 4.4. Atomic Force Microscopy

Further visualization of Borrelia biofilm structure after antimicrobial treatments (Thermos Scientific, Waltham, MA, USA) were performed using atomic force microscopy. BSK-H media was removed immediately before biofilms were analyzed. All scans were conducted using contact mode AFM imaging in air using the Nanosurf Easyscan 2 AFM (Nanosurf, Woburn, MA, USA) using SHOCONG probes (AppNANO, Mountain View, CA, USA) Images were processed using Gwyddion software (Department of Nanometrology, Czech Metrology Institute. Brno, Czech Republic) [67].

### 4.5. Statistical Analysis

Quantitative results were analyzed using the median value of all the readings from antimicrobial screen in addition to a two-tailed Student’s *t*-test (Microsoft Excel, Redmond, WA, USA) as well as graphed using Microsoft Excel software. All experiments were performed a minimum of four independent times with at least three samples per experiment. Data represents the mean ± SD.

## 5. Conclusions 

In conclusion, the findings from this study showed that whole bee venom and melittin were effective against all *B. burgdorferi* morphological forms in vitro, including antibiotic resistant attached biofilms. Though the findings from this in vitro study cannot be applied directly to clinical practice, it gives insight into the potential use of bee venom and its components against *B. burgdorferi.*

## Figures and Tables

**Table 1:** The effects of various antimicrobial agents on *B. burgdorferi* as determined by SYBR Green I/PI assay (Panel (A)) or direct counting assay (Panel (B)). Doxycycline, Cefoperazone, Daptomycin and their combination (D + C + D) as well as different concentration of bee venom and melittin were tested on *B. burgdorferi* logarithmic phase (spirochetes) culture and stationary phase (persisters) cultures as well as in 7-day recovery subculture as described previously [6,7,8,26]. *n* = 9.

**A. SYBR Green I/PI Assay**	**Spirochetes**			**Persisters**			**7 Day Subculture**		
**Treatments**	**% Control**	**% SD**	**% Median**	**% Control**	**% SD**	**% Median**	**% Control**	**% SD**	**% Median**
**Control**	100	11	100	100	12	100	100	16	100
**Doxycycline (10 μg/mL)**	33	4	33	83	3	86	122	2	83
**Cefoperazone (10 μg/mL)**	67	1	66	38	22	41	4	9	3
**Daptomycin (10 μg/mL)**	108	2	107	113	32	120	52	8	43
**D + C + D (10 μg/mL)**	66	1	65	32	7	34	3	9	2
**Bee venom (100 μg/mL)**	61	11	63	62	7	74	88	29	141
**Bee venom (400 μg/mL)**	45	11	37	44	25	43	59	19	115
**Bee venom (800 μg/mL)**	33	8	32	32	2	35	95	31	55
**Melittin (50 μg/mL)**	65	1	67	105	6	91	113	3	103
**Melittin (200 μg/mL)**	65	24	67	106	0	94	75	0	69
**Melittin (400 μg/mL)**	49	6	54	61	2	57	87	1	80
**B. Direct Counting Assay**	**Spirochetes**			**Persisters**			**7 Day Subculture**		
**Treatments**	**% Control**	**% SD**	**% Median**	**% Control**	**% SD**	**% Median**	**% Control**	**% SD**	**% Median**
**Control**	100	19	100	100	5	100	100	8	100
**Doxycycline (10 μg/mL)**	5	6	5	62	10	67	72	12	60
**Cefoperazone (10 μg/mL)**	1	1	0	43	4	43	2	6	2
**Daptomycin (10 μg/mL)**	65	7	73	34	5	36	35	6	175
**D + C + D (10 μg/mL)**	0	0	0	17	3	17	2	6	0
**Bee venom (100 μg/mL)**	5	6	4	80	10	79	79	5	65
**Bee venom (400 μg/mL)**	0	0	0	22	4	21	47	2	29
**Bee venom (800 μg/mL)**	0	0	0	8	3	7	1	1	0
**Melittin (50 μg/mL)**	0	0	0	13	5	13	0	0	0
**Melittin (200 μg/mL)**	0	0	0	0	0	0	0	0	0
**Melittin (400 μg/mL)**	0	0	0	0	0	0	0	0	0

## Supplementary Materials

The following are available online at www.mdpi.com/2079-6382/6/4/31/s1, Figure S1: The single dose effects of various antimicrobial agents on *B. burgdorferi* for as determined by SYBR Green I/PI assay (Panel A) or direct counting assay (Panel B). Figure S2: Representative Live/Dead staining images of *B. burgdorferi* log phase spirochetal cultures following single dose treatment with different antimicrobial agents. Figure S3: Representative Live/Dead staining images of *B. burgdorferi* stationary phase persister cultures following single dose treatment with different antimicrobial agents Figure S4: Representative Live/Dead staining images of *B. burgdorferi* 7-day recovery cultures following single treatment with different antimicrobial agents. Table S1: The single dose effects of various antimicrobial agents on *B. burgdorferi* as determined by SYBR Green I/PI assay (Panel A) or direct counting assay (Panel B).
