# Supplementary material for: Antimicrobial Activity of Bee Venom and Melittin against Borrelia burgdorferi
Source: Antibiotics (Basel). 2017 Nov 29;6(4):31. doi: 10.3390/antibiotics6040031 (PMC5745474; doi:10.3390/antibiotics6040031)
Supplement: Supplementary file 1 [file antibiotics-06-00031-s001.pdf]

Article

Antimicrobial Activity of Bee Venom and Melittin against Borrelia Burgdorferi

Kayla M. Socarras, Priyanka A. S. Theophilus, Jason P. Torres, Khusali Gupta and Eva Sapi *

Supplementary Materials

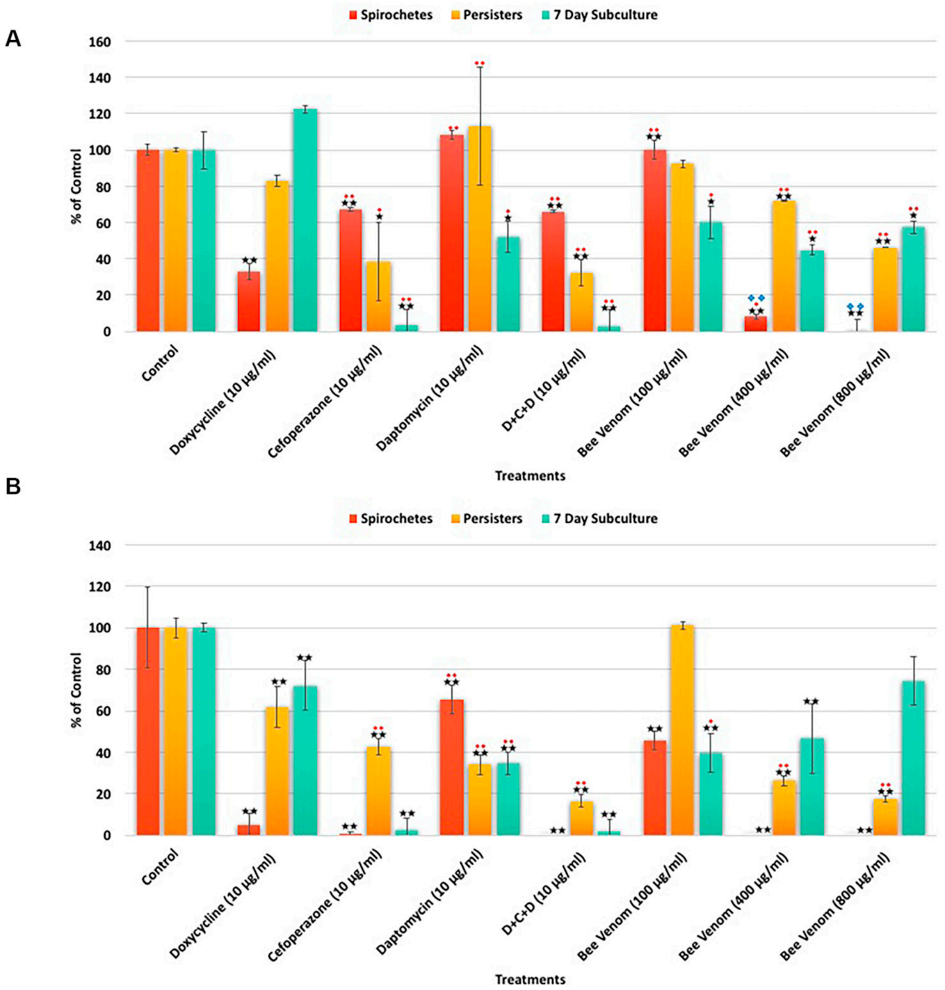

Figure S1. The single dose effects of various antimicrobial agents on *B. burgdorferi* for as determined by SYBR Green I/PI assay (Panel A) or direct counting assay (Panel B). Doxycycline, Cefoperazone, Daptomycin, and their combination (D+C+D) as well as different concentration of bee venom and melittin were tested on *B. burgdorferi* logarithmic phase (spirochetes) culture and stationary phase (persisters) cultures as well as in 7-day recovery subculture as described previously. Significance against PBS buffer (control vehicle) with the p value of < 0.05 and < 0.01 are indicated in ★ and ★★ respectively. Significance against Doxycycline with the p value of < 0.05 and < 0.01 are respectively indicated in ♦ and ♦♦ Significance against the three-antibiotic combination D+C+D with the *p*-value *of* < 0.05 and < 0.01 are indicated in ❖ and ❖❖ respectively. N=9

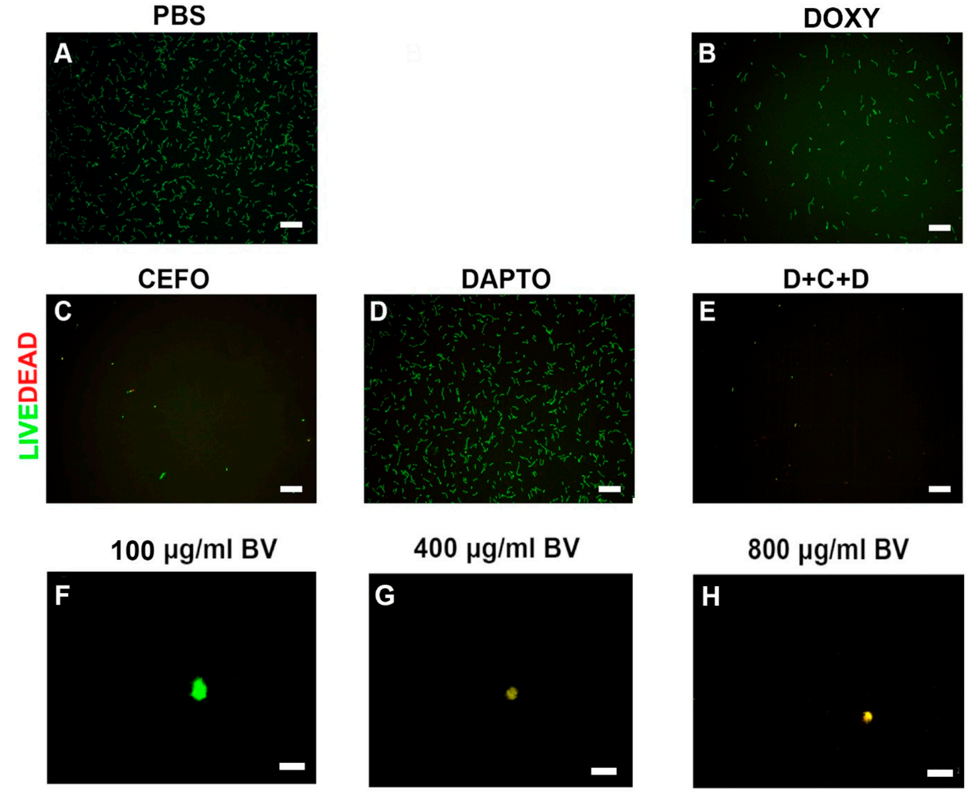

Figure S2. Representative Live/Dead staining images of *B. burgdorferi* log phase spirochetal cultures following single dose treatment with different antimicrobial agents. Cells were stained with SYBR Green I/PI as outlined in the Methods and representative images were taken at 100× magnification. (A) Borrelia culture treated only with PBS was used as a negative control. Panel B: Doxycycline (DOXY) treated, Panel C: Cefoperazone (CEFO) treated, Panel D: Daptomycin (DAPTO) treated and Panel E: Three-antibiotic combination (D+C+D) treated. Panels F-H: Bee venom (BV) was used in increasing concentrations. Live cells are stained with green color while dead cells are stained red. Scale bar:100 µm.

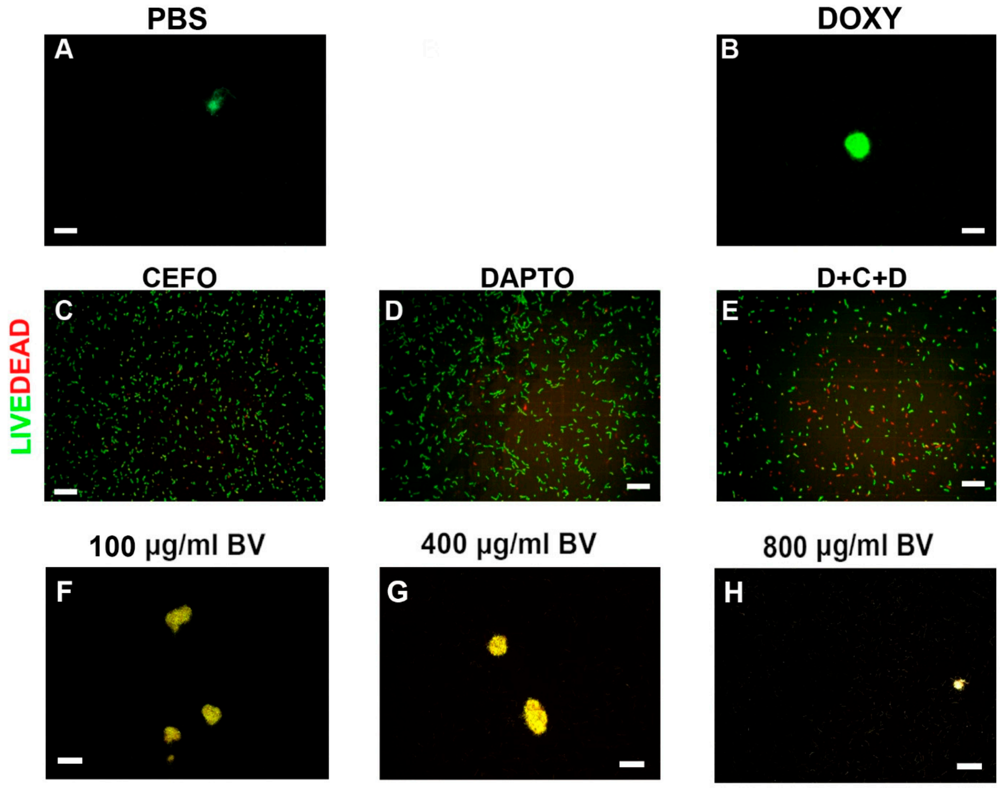

Figure S3. Representative Live/Dead staining images of *B. burgdorferi* stationary phase persister cultures following single dose treatment with different antimicrobial agents. Cells were stained with SYBR Green I/PI as outlined in the Methods and representative images were taken at 100× magnification. (A) Borrelia culture treated only with PBS was used as a negative control. Panel B: Doxycycline (DOXY) treated, Panel C: Cefoperazone (CEFO) treated, Panel D: Daptomycin (DAPTO) treated and Panel E: Three-antibiotic combination (D+C+D) treatment. Panels F-H: Bee venom (BV) was used in increasing concentrations. Live cells are stained with green color while dead cells are stained red. Scale bar: 100 µm.

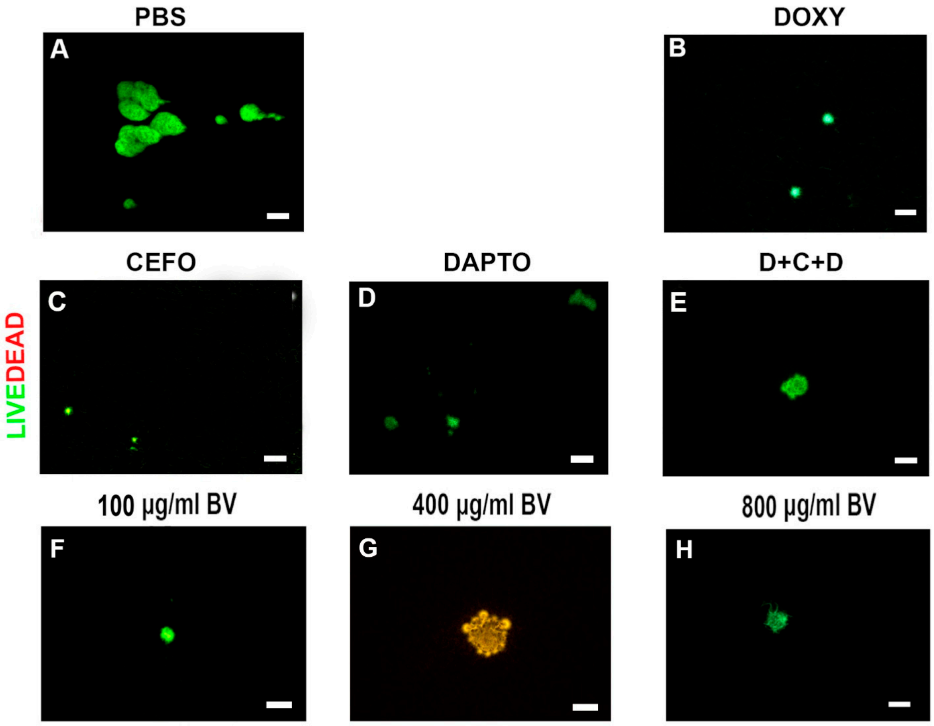

Figure S4. Representative Live/Dead staining images of *B. burgdorferi* 7-day recovery cultures following single treatment with different antimicrobial agents. Cells were stained with SYBR Green I/PI as outlined in the Material and Methods and representative images were taken at 100× magnification. (A) Borrelia culture treated only with PBS was used as a negative control. Panel B: Doxycycline (DOXY) treated, Panel C: Cefoperazone (CEFO) treated, Panel D: Daptomycin (DAPTO) treated and Panel E: Three-antibiotic combination (D+C+D) treatment. Panels F-H: Bee venom (BV) was used in increasing concentrations. Live cells are stained with green color while dead cells are stained red. Scale bar: 100 µm.

Table S1. The single dose effects of various antimicrobial agents on *B. burgdorferi* as determined by SYBR Green I/PI assay (Panel A) or direct counting assay (Panel B). Doxycycline, Cefoperazone, Daptomycin, and their combination (D+C+D) as well as different concentrations of bee venom and melittin were tested on *B. burgdorferi* logarithmic phase (spirochetes) culture and stationary phase (persisters) cultures as well as in 7-day recovery subculture as described previously [6, 7, 8, 26]. N=9

**A. SYBR Green I / PI assay**	**Spirochetes**			**Persisters**			**7 Day Subculture**		
**Treatments**	**% Control**	**% SD**	**% Median**	**% Control**	**% SD**	**% Median**	**% Control**	**% SD**	**% Median**
Control	100	11	100	100	12	100	100	16	100
Doxycycline (10 µg/ml)	33	4	33	83	3	86	122	2	83
Cefoperazone (10 µg/ml)	67	1	66	38	22	41	4	9	3
Daptomycin (10 µg/ml)	108	2	107	113	32	120	52	8	43
D+C+D (10 µg/ml)	66	1	65	32	7	34	3	9	2
Bee venom (100 µg/ml)	60.6	11.2	62	96	7	98	88	29	103
Bee venom (400 µg/ml)	44.9	11.5	44	68	25	79	59	19	87
Bee venom (800 µg/ml)	33.2	8.0	41	53	2	55	95	31	104

**B. Direct Counting Assay**	**Spirochetes**			**Persisters**			**7 Day Subculture**		
**Treatments**	**% Control**	**% SD**	**% Median**	**% Control**	**% SD**	**% Median**	**% Control**	**% SD**	**% Median**
Control	100	19	100	100	5	100	100	8	100
Doxycycline (10 µg/ml)	5	6	5	62	10	67	72	12	60
Cefoperazone (10 µg/ml)	1	1	0	43	4	43	2	6	2
Daptomycin (10 µg/ml)	65	7	73	34	5	36	35	6	175
D+C+D (10 µg/ml)	0	0	0	17	3	17	2	6	0
Bee venom (100 µg/ml)	46	4	46	101	2	101	31	7	34
Bee venom (400 µg/ml)	0	0	0	26	2	26	36	13	37
Bee venom (800 µg/ml)	0	0	0	17	1	17	58	9	58

© 2017 by the authors. Submitted for possible open access publication under the terms and conditions of the Creative Commons Attribution (CC BY) license (http://creativecommons.org/licenses/by/4.0/).
